# Effect of Strengthening Mechanism of Alkali Curing on Mechanical Properties of Fly Ash Lightweight Aggregates and Its Concrete

**DOI:** 10.3390/ma18010089

**Published:** 2024-12-28

**Authors:** Jun Liu, Zhishan Xu, Yongsheng Ji

**Affiliations:** 1 Department of Environmental Art Engineering, Nanjing Technical Vocational College, Nanjing 210037, China; 2 College of Wood Science and Technology, Nanjing Forestry University, Nanjing 210037, China; 3 School of Civil and Harbor Engineering, Jiangsu Ocean University, Lianyungang 222005, China; 4 Jiangsu Key Laboratory Environmental Impact and Structural Safety in Engineering, China University of Mining and Technology, Xuzhou 221116, China; jiyongsheng@cumt.edu.cn

**Keywords:** Fly Ash Unburned Lightweight Aggregate, alkali curing, concrete, interfacial transition zone, microhardness

## Abstract

The low hydration degree of fly ash in Fly Ash Unburned Lightweight Aggregate (FULA) is not conducive to the development of the mechanical properties of lightweight aggregates and their concrete. In this paper, FULA was immersed in an alkaline solution with the purpose of improving the mechanical properties of FULA and its concrete. Firstly, FULA was prepared using fly ash as the main raw material. The effect of the alkaline solution type and concentration on the basic properties of FULA was studied. Then, lightweight aggregate concrete was prepared using FULA as a coarse aggregate. The role of the aggregate category and water–cement ratio in the mechanical properties of concrete was analyzed. Finally, the effect of alkali curing on the interfacial transition zone of concrete was tested by combining an electron microscope and microhardness tester. Based on this, the strengthening mechanism of alkali curing on the mechanical properties of concrete was proposed. The results show that alkali curing can effectively improve the mechanical properties of FULA and its concrete. The microhardness of the interfacial transition zone of alkali curing FULA concrete is significantly higher than that of the cementite matrix, and the closer the aggregate, the higher the microhardness. The fundamental reason for this is that alkali curing improves the hydration degree of fly ash.

## 1. Introduction

Sand and gravel aggregate accounts for 70–80% of the quality of concrete and is the second largest resource used by humans after water [1,2,3]. At present, China’s annual consumption of sand and gravel aggregate is about 20 billion tons, accounting for about one-half of the world’s total annual production and sales of sand and gravel aggregate. However, with the introduction of low-carbon, environmental protection, and sustainable development policies, a large number of sand and gravel plants have been shut down and reorganized. The imbalance between supply and demand makes the price of sand and gravel aggregate rise sharply. At the same time, industrial waste fly ash annual production is huge, and the degree of resource utilization is low. It is very easy to cause serious adverse effects on the environment [4,5]. In the context of the long-term high price of sand and gravel and the deep development and utilization of solid waste building materials, the use of alternative materials has become increasingly important. The preparation of lightweight aggregates with fly ash to replace sand and gravel has become one of the important trends in the building materials industry [6,7,8].

At present, the preparation methods of lightweight aggregates mainly include the roasting method and no-burning method [9,10]. The roasting method consumes a lot of energy. This method also pollutes the environment, which is not conducive to the development of lightweight aggregates [11]. The no-burn method is a simple process with low energy consumption, which is conducive to promoting the utilization of solid waste as building materials [11]. According to the difference in cementing principles, the no-burn lightweight aggregate is mainly divided into a cement lightweight aggregate and a geopolymer lightweight aggregate. For example, in fly ash mixed with a small amount of cement, FULA is manufactured by relying on cement-prepared products with a certain mechanical strength [12,13,14]. In fly ash mixed with a certain amount of alkaline activator, alkali-activated FULA is manufactured using a complex polymerization reaction prepared with a certain mechanical strength of the product [15,16,17]. Studies have shown that these two kinds of FULA are characterized by light weight, low density, and high porosity. They have a wide range of application prospects in light aggregate concrete, filter filler, sound insulation boards, and so on [18,19,20].

However, the higher content of fly ash in cement FULA and the lower mechanical strength after curing are detrimental to the development of the mechanical properties of lightweight aggregates and their concrete [21]. The reason is that the activity of fly ash is low, and it is difficult to fully hydrate after mixing with cement [22]. For this reason, some researchers have drawn on the geopolymer preparation method in fly ash mixed with alkaline activator to improve its activity and successfully prepared alkali-activated FULA with high mechanical strength [23,24]. However, alkali-activated FULA has the following defects: the reaction happens too quickly, the alkalinity is too high, and it is difficult to make alkali-activated FULA compatible with cementitious materials [25,26,27]. As a result, the application of alkali-activated FULA in cementitious materials has been seriously limited. Consider that the curing period after the molding of cement FULA is long. Therefore, if it is soaked and maintained with an alkali activator during the curing period, it may be possible to both improve the hydration degree of fly ash and avoid the defects of alkali-activated FULA. Meanwhile, the alkaline solution can be recycled, which is more economical and environmentally friendly than alkali-activated FULA.

In this paper, a reinforcement method is proposed to improve the mechanical properties of FULA and its concrete by soaking and curing cement FULA (all subsequent FULA refers to unburned lightweight aggregate with cement as the binder) in an alkaline solution. First, the effects of the cement content on the basic properties of FULA were investigated. Then, the effects of the alkaline solution on the basic properties and physical phase composition of FULA were investigated. Finally, lightweight aggregate concrete was prepared using FULA as a coarse aggregate, and the effects of the lightweight aggregate type and water–cement ratio on the mechanical properties of concrete were investigated. Additionally, the construction principle of the “alkali-conditioned FULA-cement” interfacial transition zone (ITZ) in concrete was analyzed. Based on these findings, the strengthening mechanism of alkali curing on the mechanical properties of FULA and its concrete was proposed.

## 2. Materials and Methods

### 2.1. Materials

#### 2.1.1. Cement

Ordinary Portland cement (OC, Grade 42.5) is utilized as the binder for FULA. The cement has a surface area of 330 m^2^/kg and a specific gravity of 3.14 g/cm^3^. The standard consistency water demand and fineness (0.08 mm Square sieve) are 28.1% and 1.02%. Its mineral and chemical composition are given in Table 1 and Table 2, respectively.

#### 2.1.2. Fly Ash

Low-quality tertiary fly ash, provided by Xuzhou Maocun Thermal Power Plant (Xuzhou, China), serves as the primary raw material for the preparation of FULA. The fineness (0.045 mm sieve residue) and water requirement of the fly ash are 89% and 112%, respectively. The loss on ignition and water content are 4.35% and 28.7%, respectively (dried when used). The average particle size is 8.2 microns. The specific chemical composition is shown in Table 2.

#### 2.1.3. Alkaline Curing Solution

The alkaline curing solution for FULA is prepared using the chemical reagents sodium hydroxide (NaOH), sodium chloride (NaCl) and sodium sulfate (Na_2_SO_4_). The purity of sodium hydroxide is 96%, the density is 2.13 g/cm^3^, and the solubility is 109 g at 20 °C. The purity of sodium chloride is 97%, the density is 2.165 g/cm^3^, and the solubility is 35.9 g at 20 °C. The purity of sodium sulfate is 99.5%, the density is 2.68 g/cm^3^, and the solubility at 20 °C is 19.5 g.

#### 2.1.4. Others

River sand, with a fineness modulus of 2.61, is utilized as the fine aggregate for concrete. Continuously graded gravel, with particle sizes ranging from 5 to 20 mm, along with ordinary FULA and alkali-cured FULA, are used as the coarse aggregates in concrete. The additive employed is poly-carboxylic acid high-performance water-reducing agent, produced by Xuzhou Xingu Building Material Technology Co., Ltd. (Xuzhou, China), which boasts a water-reducing rate of over 25%. The test water utilized is tap water.

### 2.2. Preparation of Lightweight Aggregates and Its Concrete

#### 2.2.1. Preparation of FULA

**Figure 1:**
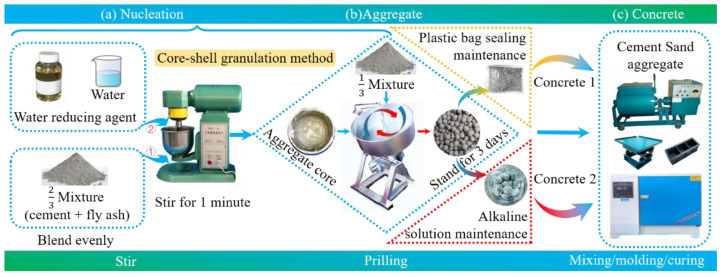
Preparation of lightweight aggregate and its concrete.

Figure 1 shows the preparation of lightweight aggregate and its concrete. Firstly, fly ash and cement are homogenized in proportion to form a mixture. Take 2/3 mass of the mixture with water and water reducing agent and mix (about 1 min) to form agglomerates (aggregate cores). Add 1/3 mass of the mixture with agglomerates to a disk granulator (PQ(T)-500, manufactured by Zhengzhou Mining Machinery Co., Ltd., Zhengzhou, China). At a rotational speed of 40 r/min (running for about 20 min), the agglomerates are caused to move parabolically along the disk, and FULA of different sizes are gradually formed. The formed FULA is taken out and left to stand for 3 days. One part of the FULA is sealed in a plastic bag (normal FULA), and the other part is poured into an alkaline solution for soaking (alkali curing FULA). All FULAs are removed and dried when they reach the age designated for measurement.

#### 2.2.2. Preparation of FULA Concrete

Normal lightweight aggregate concrete is prepared using FULA that has been cured in sealed plastic bags. Alkali curing lightweight aggregate concrete is prepared using FULA that has been cured in alkaline solution. All lightweight aggregate concretes are vibrated and molded in 100 mm × 100 mm × 100 mm triple molds. Following molding, the concrete is placed in a standard curing box (temperature of 20 ± 2 °C and relative humidity ≥ 95%) for curing. Demolding occurs after one day, and subsequently, the standard curing process continues until the specified age is reached.

### 2.3. Methods

#### 2.3.1. Effect of Cement Content on the Basic Properties of Ordinary FULA

FULA was prepared following the procedure outlined in Section 2.2. Fly ash and cement were mixed as a mixture. The core of the FULA was prepared using two-thirds of the mixture’s mass (the remaining one-third was utilized for preparing the shell of the FULA). The water-to-cement ratio employed was 0.2, and the water-reducing agent was added at a dosage of 1.0% of the mixture’s mass. Cement content was 10%, 20%, 30%, and 40% of the mixture. The FULAs were numbered A1, A2, A3, and A4 in that order. The maintenance was taken out and dried at the age of 28 days. According to GB/T 17431.2-2010 [28], the numerical tube pressure, 1 h water absorption and packing density of FULA were tested. The FULA with the optimal performance and its corresponding mix ratio were identified.

#### 2.3.2. Effect of Alkali Curing on the Basic Properties of FULA

(1) Effect of Curing Solution Type on the Numerical Tube Pressure of FULA

Seven groups of FULA, designated as B0, B1, …, and B6, were prepared based on the preferred ratios of lightweight aggregates outlined in Section 2.3.1. Group B0 served as a control group, cured within sealed plastic bags. Groups B1 through B3 were individually cured using single reagents: 10% concentration solutions of NaOH, NaCl, and Na_2_SO_4_, respectively. Groups B4 to B6 utilized composite exciters consisting of 10% concentration solutions where each component (NaOH + NaCl, NaOH + Na_2_SO_4_, and NaCl + Na_2_SO_4_) was maintained at a 5% concentration for each individual exciter. At the curing ages of 7, 14, and 28 days (calculated as the sum of resting and soaking times), the cylinders were retrieved, dried, and subsequently tested for the numerical tube pressure.

(2) Effect of Curing Solution Concentration on the Basic Properties of FULA

Five groups of FULA, designated as C0, C1, C2, C3, and C4, were prepared in accordance with the preferred lightweight aggregate conservation solution outlined in Section 2.3.2 (1). Group C0 served as a control group, undergoing sealed curing within plastic bags. The curing solutions for Groups C1 through C4 were formulated at concentrations of 10%, 20%, 30%, and 40%, respectively. After reaching a curing age of 28 days, the specimens were removed for drying. Subsequently, they were tested for numerical tube pressure, 1 h water absorption, and packing density.

#### 2.3.3. Effect of Alkali Curing on the Physical Phase Composition of FULA

Took a small amount of C0, C3 & C4 light aggregates that were cured to an age of 28 days. Dried and ground to a powder with a particle size of not more than 200 mesh. The composition of the phases and the molecular structure of the hydration products were measured by X-ray diffractometer (D8 Advance, BRUKER AXS GMBH, Karlsrühe, Germany) and infrared spectrometer (VERTEX 80v, BRUKER AXS GMBH, Karlsrühe, Germany), respectively.

#### 2.3.4. Effect of Alkali Curing FULA on Mechanical Properties of Concrete

(1) Effect of Aggregate Type on Mechanical Properties of Concrete

Three groups of concrete specimens were prepared with 0.3 water–cement ratio. The water reducing agent mixing amount was 0.5% of cement mass. The specimens were numbered as D1, D2 and D3 in sequence, D1 with gravel as coarse aggregate, D2 with FULA as coarse aggregate and D3 with alkali curing FULA as coarse aggregate, and the specific mix ratios are shown in Table 3. The volume of coarse aggregate used was the same for all three sets of concrete. The FULA and alkali curing FULA were wetted to a saturated surface dry condition before mixing the concrete. When the specimens were molded and cured to the age of 3, 7, and 28 days, their compressive strength was measured according to GB/T 50081-2019 [29].

(2) Effect of Water–Cement Ratio on Mechanical Properties of Concrete

Four groups of specimens were prepared utilizing the preferred lightweight aggregates and their corresponding concrete mix ratios as specified in Section 2.3.4 (1). The concentration of the water reducer remained constant at 0.5% of the cement mass. The specimens, numbered E1, E2, E3, and E4 in sequence, were produced with water–cement ratios of 0.3, 0.35, 0.4, and 0.45, respectively. The compressive strength of these specimens was evaluated after they were cast and cured for durations of 3, 7, and 28 days.

#### 2.3.5. Effect of Alkali Curing FULA on the Microstructure of the Interfacial Transition Zone

(1) Effect of Alkali Curing FULA on Microhardness of Interfacial Transition Zone

Seven groups of concrete specimens were prepared in accordance with the mix ratios outlined in Section 2.3.4 (1) and (2). The concrete was cast into cylindrical molds with dimensions of φ30 mm × 100 mm. After one day of curing, the specimens were demolded and subsequently underwent standardized curing until they reached 28 days of age. To investigate the microhardness of the interface transition zone, a cutting machine was utilized to obtain thin slices, approximately 15 mm thick, from the central portion of each specimen. Following polishing and grinding, the micro-Vickers hardness tester (model HVS-1000, Fuguang precision instrument co., ltd, Shanghai, China) was employed to measure the hardness. Indentations were made within a 180 μm distance from the aggregate surface to the cementitious matrix, and tests were conducted at intervals of 20 μm. At each indentation point, an average of 10 measurements was taken.

(2) Effect of Alkali Curing FULA on the Micro-Morphology of the Interfacial Transition Zone

Scanning electron microscope (FEI Quanta TM 250, FEI Company, Hillsboro, OR, USA) was used to analyze the D1, D2 and D3 specimens at the age of 28 days of curing. The micro-morphology of the transition zone at the aggregate-cementite interface was observed.

## 3. Results and Discussion

### 3.1. Ordinary FULA Basic Performance Analysis

**Figure 2:**
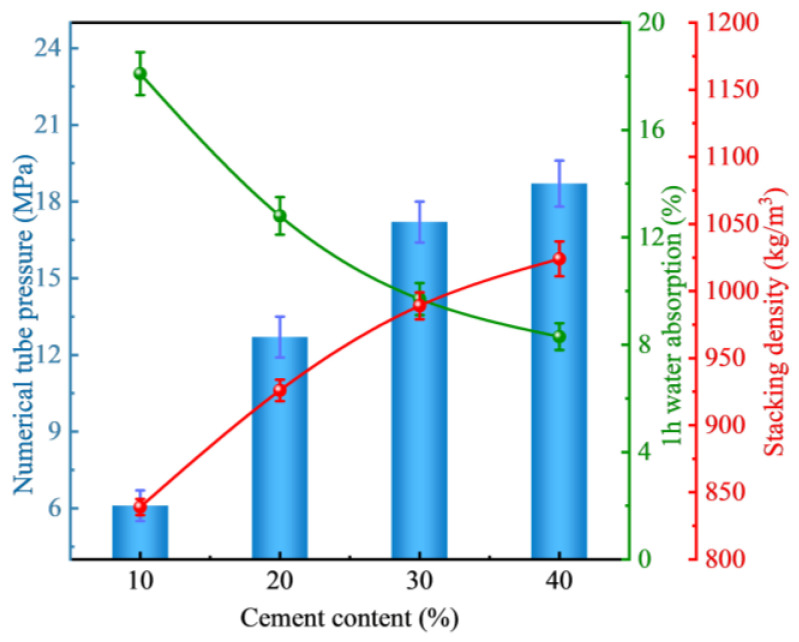
Effect of cement content on the basic properties of FULA.

Figure 2 illustrates the impact of cement content on the fundamental properties of FULA. Upon examining the figure, it is evident that as the cement content increases from 10% to 20%, the numerical tube pressure of FULA rises from 6.0 MPa to 12.7 MPa, marking an increase of 111.67%. Similarly, when the cement content is elevated to 30%, the numerical tube pressure further increases to 17.1 MPa, which is a 34.65% augmentation compared to the 20% cement content. At a cement content of 40%, the numerical tube pressure reaches 18.7 MPa, representing a 9.61% enhancement over the 30% cement content. These observations suggest that an increase in cement content leads to a corresponding increase in the numerical tube pressure of FULA. This trend can be attributed to cement’s higher reactivity compared to fly ash, as well as the increased formation of hydration products with higher cement content. Additionally, the calcium hydroxide produced during cement hydration facilitates the conversion of fly ash into hydrated calcium silicate.

**Figure 3:**
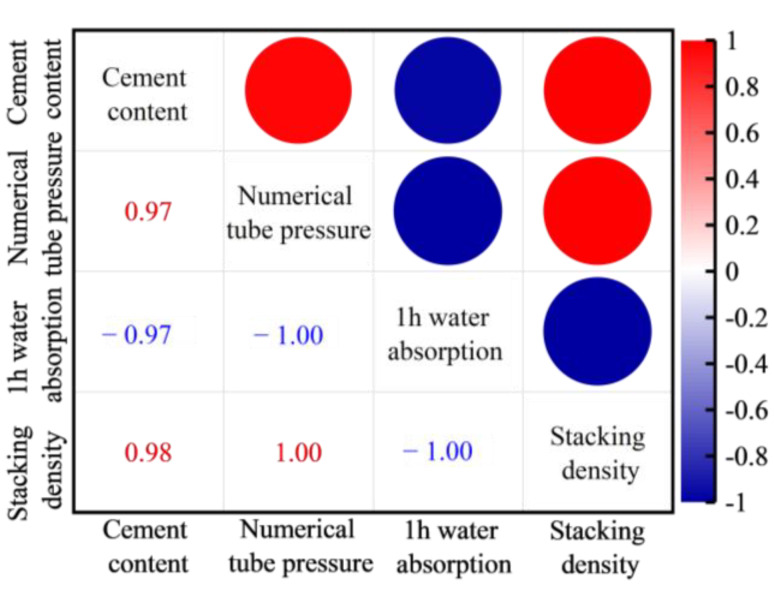
Correlation between cement content and basic properties of FULA.

Similarly, the packing density of FULA exhibits an upward trend, increasing from 840 kg/m^3^ to 1023 kg/m^3^ as the cement content rises from 10% to 40%. Conversely, the 1-h water absorption of FULA demonstrates a notable decline, decreasing from 18.3% to 8.4%, with increasing cement content. Specifically, Figure 3 [30,31] illustrates the correlations between cement content and the fundamental properties of FULA, revealing that cement content is positively correlated with both numerical tube pressure (r = 0.97) and packing density (r = 0.98), while it is negatively correlated with 1-h water absorption (r = −0.97). This trend may be attributed to the enhanced densification of FULA due to increased cement content. Given the low-carbon benefits and economic considerations, FULA can achieve satisfactory molding and establish adequate mechanical properties with a cement dosage of 10%. Consequently, subsequent experiments were conducted using FULA prepared with 10% cement content.

### 3.2. Alkali Curing FULA Basic Performance Analysis

#### 3.2.1. Types of Curing Solution

**Figure 4:**
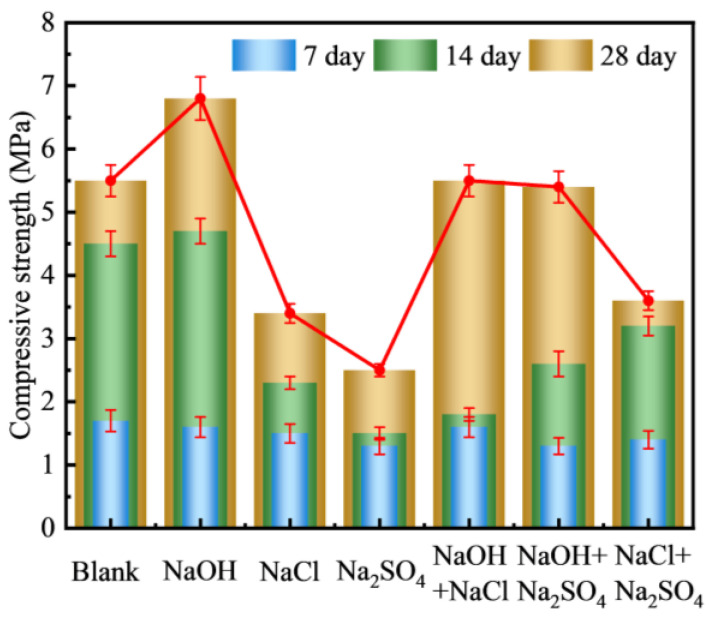
Effect of curing solution types on the numerical tube pressure of FULA.

Figure 4 shows the effect of different alkaline curing solution on the numerical tube pressure of FULA. From the figure, comparing with the blank group, the effect of alkaline curing on the numerical tube pressure of FULA at early age (7 days) is not obvious, and the effect on the long age is more significant. Specifically, the numerical tube pressure of FULA in group 7 at 7 days of curing was 1.50 ± 0.5 MPa. At 14/28 days of curing, the numerical tube pressure of FULA cured with sodium hydroxide solution is higher than that of the blank group. While the numerical tube pressure of FULA cured with sodium chloride, sodium sulfate, and composite solutions (NaOH + NaCl, NaOH + Na_2_SO_4_, NaCl + Na_2_SO_4_) is less than that of the blank group.

The results indicate that sodium hydroxide has a positive effect on enhancing the numerical tube pressure of FULA, whereas sodium chloride, sodium sulfate, and their composite solutions are detrimental to the development of this pressure. This may be attributed to the alkaline and ionic environment provided by the sodium hydroxide solution, which is more favorable for promoting the hydration of fly ash and the formation of a favorable microstructure within FULA.

**Figure 5:**
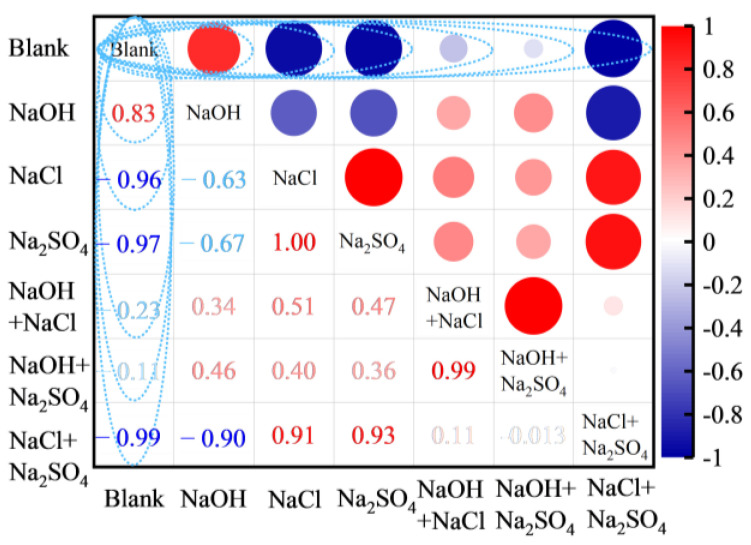
Correlation between alkali solution and numerical tube pressure based on blank group.

To quantify the impact of various alkaline solutions on the numerical tube pressure of FULA, a heat map was generated, depicting the correlation between these solutions and the numerical tube pressure, with the blank group serving as a benchmark. As illustrated in Figure 5, the correlations between the numerical tube pressure of FULA and the conditioning solutions of sodium hydroxide, sodium chloride, and sodium sulfate are positive (0.83), negative (−0.96), and negative (−0.97), respectively. Similarly, the correlations with the composite solutions of “NaOH + NaCl,” “NaOH + Na_2_SO_4_,” and “NaCl + Na_2_SO_4_” are negative (−0.23), negative (−0.96), and negative (−0.97), respectively. These findings suggest that a sodium hydroxide conservation solution can effectively stimulate the reactivity of fly ash, whereas sodium chloride and sodium sulfate solutions hinder the hydration process of fly ash. Consequently, sodium hydroxide solution was selected for subsequent alkaline curing tests of FULA.

#### 3.2.2. Concentration of Curing Solution

**Figure 6:**
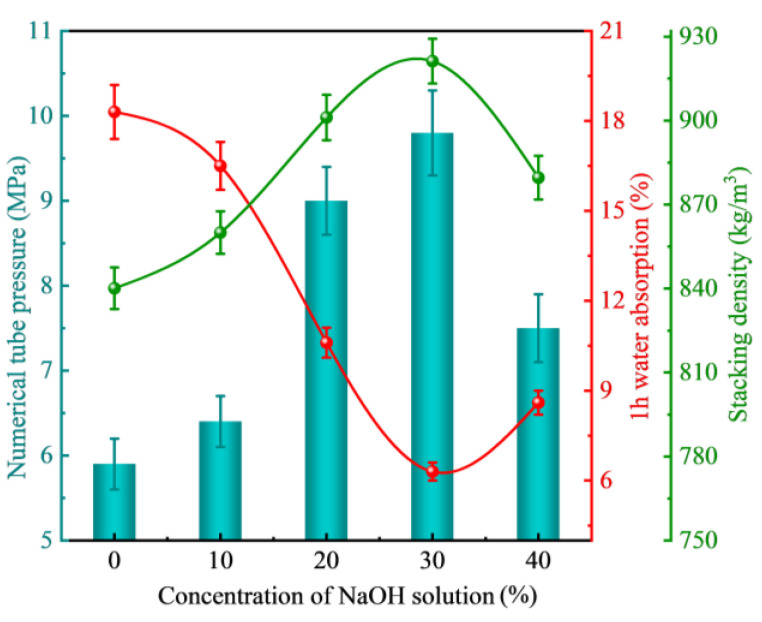
Effect of alkali solution concentration on the basic properties of FULA.

Figure 6 illustrates the influence of sodium hydroxide curing solution concentration on the fundamental properties of FULA after 28 days of aging. Upon examination of the figure, it becomes apparent that as the concentration of the curing solution increases, the numerical tube pressure of FULA exhibits an initial increase followed by a decline. Specifically, when the curing solution concentration reaches 30%, the numerical tube pressure of FULA attains a peak value of 9.71 MPa, representing a 62% enhancement compared to the blank group.

This finding suggests that an increase in the concentration of the sodium hydroxide curing solution can be beneficial for enhancing the numerical tube pressure of FULA, however, excessively high concentrations are detrimental to its further development. One plausible explanation for this observation is that higher concentrations of the sodium hydroxide curing solution may destabilize the hydration products, leading to a decrease in the numerical tube pressure of FULA.

### 3.3. Physical Composition Analysis of Alkali Curing FULA

#### 3.3.1. XRD

**Figure 7:**
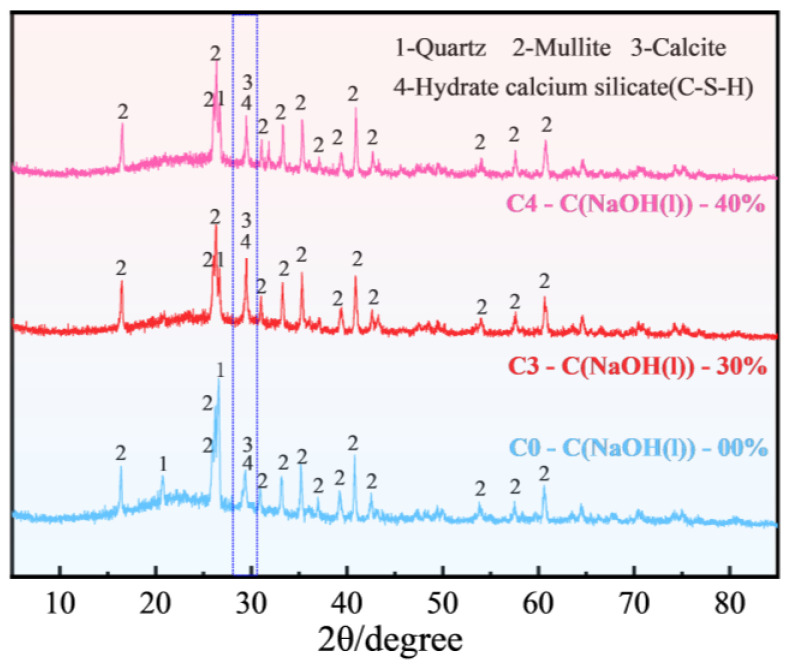
XRD of FULA.

Figure 7 shows the XRD patterns of FULA treated with different concentrations of sodium hydroxide conservation solution. From the figure, the main physical phase compositions of specimens C0, C3 and C4 are: mullite (3Al_2_O_3_-2SiO_2_), quartz (SiO_2_), calcite (CaCO_3_) and hydrated calcium silicate (C-S-H). It is known that no new minerals are produced in FULA after alkali conservation.

When comparing specimen C0 to the condition where the sodium hydroxide solution concentration is 30%, notable alterations in the diffraction peak intensities of FULA are observed. Specifically, the intensity of the quartz characteristic peak undergoes a substantial decrease, whereas the intensity of the hydrated calcium silicate characteristic peak experiences a significant increase. This suggests that a considerable amount of SiO_2_ from the fly ash is consumed, leading to the formation of numerous C-S-H gels. The underlying mechanism is that sodium hydroxide enhances the reactivity of the fly ash, which is beneficial for improving the mechanical properties of FULA.

In contrast to specimen C3, at a sodium hydroxide solution concentration of 40%, further modifications in the diffraction peak intensities of FULA are evident. Both the quartz characteristic peak and the hydrated calcium silicate characteristic peak exhibit a decreasing trend. This implies that, while an elevated concentration of sodium hydroxide solution can indeed promote the hydration of fly ash, it also results in a diminished production of C-S-H gel, ultimately causing an inversion in the mechanical properties of FULA. This observation aligns with the trend shown in Figure 6, which depicts the development pattern of FULA’s numerical tube pressure.

#### 3.3.2. FT-IR

**Figure 8:**
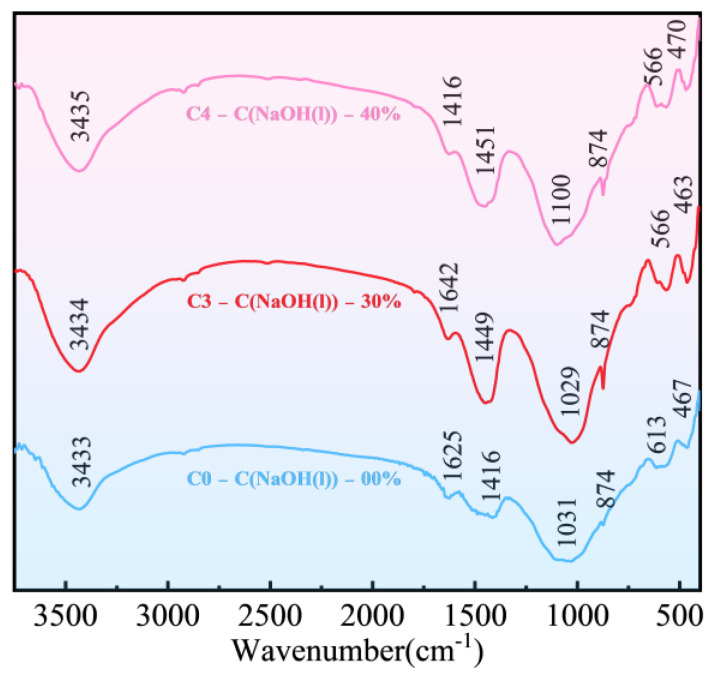
FT-IR of FULA.

Figure 8 presents the FT-IR spectra of FULA subjected to various concentrations of sodium hydroxide solution. Upon examination of the figure, samples C0, C3, and C4 all exhibit distinct IR absorption peaks at 3433 cm^−1^ (corresponding to -OH), 1625 cm^−1^ (H-OH), 1416 cm^−1^ (O-C-O), 1031 cm^−1^ (Si-O-Si), and 467 cm^−1^ (Si-O-Si). Notably, the Si-O-Si bond absorption peaks in sample C0 are relatively weaker, suggesting a lesser degree of hydration-induced calcium silicate aluminate formation. Similarly, the weaker -OH bond absorption peak indicates a lower alkaline material content in sample C0. These observations imply that the hydration level of fly ash in FULA without sodium hydroxide curing is relatively low, which underscores the challenge in achieving superior mechanical properties in conventional FULA.

Compared to specimen C0, when the sodium hydroxide solution concentration reaches 30%, the infrared absorption peaks of specimen C3 undergo more significant alterations. The absorption peak at 1029 cm^−1^ exhibits a notable increase in intensity and becomes narrower, suggesting a substantial enhancement in the formation of calcium silicate hydrate and calcium silicoaluminate hydrate within the specimen. Additionally, the absorption peak at 1449 cm^−1^ experiences a slight intensity increase, indicating the generation of substantial carbonate within the specimen. Furthermore, the absorption peak at 3434 cm^−1^ undergoes a significant intensity increase, which signifies an increased presence of alkaline material within the specimen. Consequently, the curing of FULA with a sodium hydroxide solution elevates the alkalinity of the system and facilitates the active activation of fly ash, thereby favoring the enhancement of FULA’s numerical tube pressure.

In contrast to specimen C3, when the sodium hydroxide solution concentration rises to 40%, the infrared absorption peaks of specimen C4 exhibit further changes. The intensities of the absorption peaks at 1100 cm^−1^ and 470 cm^−1^, which correspond to hydrated calcium silicate and hydrated calcium silicoaluminate, respectively, decrease slightly. Notably, the absorption peak at 1451 cm^−1^, which corresponds to the O-C-O bond, decreases in intensity rather than increasing. This observation indicates a reduction in the formation of calcium silicate hydrate, calcium silicoaluminate hydrate, and carbonate from FULA as the sodium hydroxide concentration further increases. This phenomenon serves as the underlying cause for the reversal of FULA’s mechanical properties upon further augmentation of the sodium hydroxide concentration.

### 3.4. Mechanical Properties Analysis of Alkali Curing FULA Concrete

#### 3.4.1. Types of Aggregates

**Figure 9:**
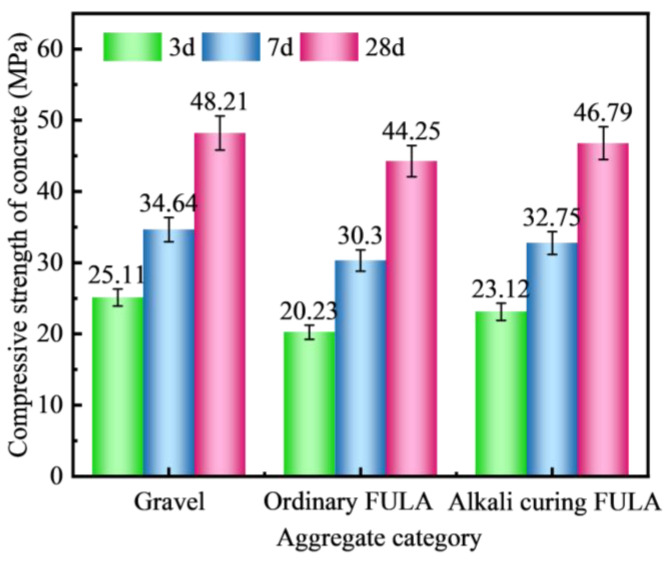
Effect of different coarse aggregates on compressive strength of concrete.

Figure 9 illustrates the impact of various aggregates on the compressive strength of concrete. At all ages of 3 days, 7 days, and 28 days, gravel concrete exhibits the highest compressive strength, achieving a peak of 48.21 MPa at 28 days. Conversely, ordinary FULA concrete demonstrates the lowest compressive strength, attaining only 44.25 MPa at 28 days. Alkali-cured FULA concrete, with a compressive strength of 46.79 MPa at 28 days, falls 2.95% below gravel concrete but surpasses ordinary FULA concrete by 5.74%.

The results indicate that the compressive strength of FULA concrete is marginally lower compared to that of ordinary concrete. However, following alkali curing, FULA can be processed to yield concrete products that exhibit higher compressive strength than ordinary FULA concrete. This enhancement may be attributed to the sodium hydroxide’s capacity to improve both the mechanical and surface properties of FULA, thereby enhancing the mechanical characteristics of alkali-cured FULA concrete.

#### 3.4.2. Water–Cement Ratio

**Figure 10:**
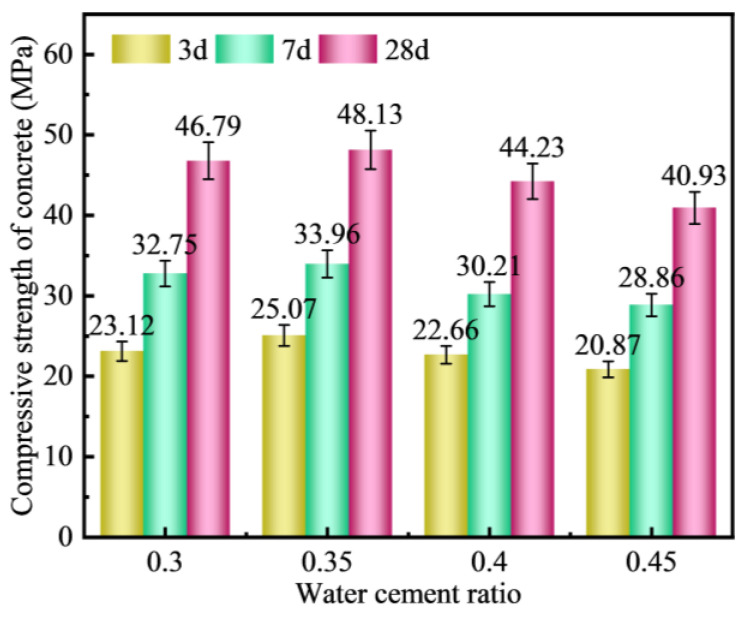
Effect of water–cement ratio on compressive strength of concrete.

Figure 10 depicts the influence of the water–cement ratio on the compressive strength of lightweight aggregate concrete. At a 3-day age, as the water–cement ratio increases, the compressive strength of the lightweight aggregate concrete initially rises from 23.12 MPa to 25.07 MPa, subsequently declining to 20.87 MPa. This pattern indicates that, with an increasing water–cement ratio, the compressive strength of the lightweight aggregate concrete at 3 days exhibits a trend of initial increase followed by a decrease.

Analogously, the compressive strength at 7 days and 28 days follows a similar trend of increasing and then decreasing. Notably, the compressive strength attains its peak value of 46.79 MPa when the water–cement ratio is 0.35.

In summary, as the water–cement ratio decreases, the compressive strength of lightweight aggregate concrete gradually increases. However, an excessively low water–cement ratio adversely affects the compressive strength. This phenomenon may be attributed to the internal curing effect of lightweight aggregates, which enhances the structure of the interfacial transition zone. When the water–cement ratio is lower, the internal curing effect of lightweight aggregates becomes more pronounced. Conversely, when the water–cement ratio is too low, although the internal curing effect continues to improve the structure of the interfacial transition zone, it compromises the molding quality of the lightweight aggregate concrete, ultimately leading to a reduction in the strength of the cementitious components in the remaining portions.

### 3.5. Analysis of the Interfacial Transition Zone of Alkali Curing FULA Concrete

#### 3.5.1. Microhardness

**Figure 11:**
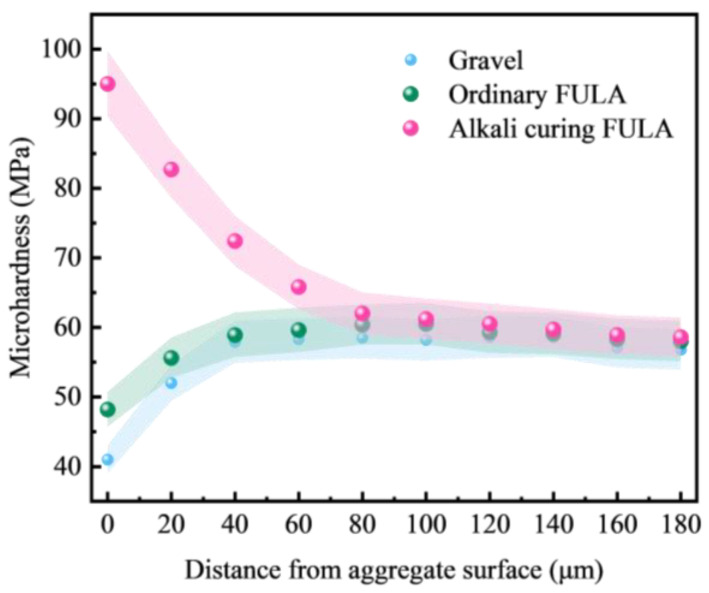
Effect of different coarse aggregates on the microhardness of interfacial transition zone.

Figure 11 illustrates the impact of various coarse aggregates on the microhardness of the interfacial transition zone (ITZ) in concrete, as reported in [32,33]. The figure reveals that the ITZ of both ordinary FULA concrete and gravel concrete exhibit similar characteristics, featuring an interfacial weak zone approximately 40 μm in width, where the microhardness is lower compared to the cement paste matrix. In stark contrast, alkali-cured FULA concrete demonstrates an interfacial enhancement zone of approximately 80 μm, with a microhardness significantly greater than that of the cement paste matrix. This observation underscores the notable effect of alkali curing on substantially elevating the microhardness of the ITZ in concrete.

**Figure 12:**
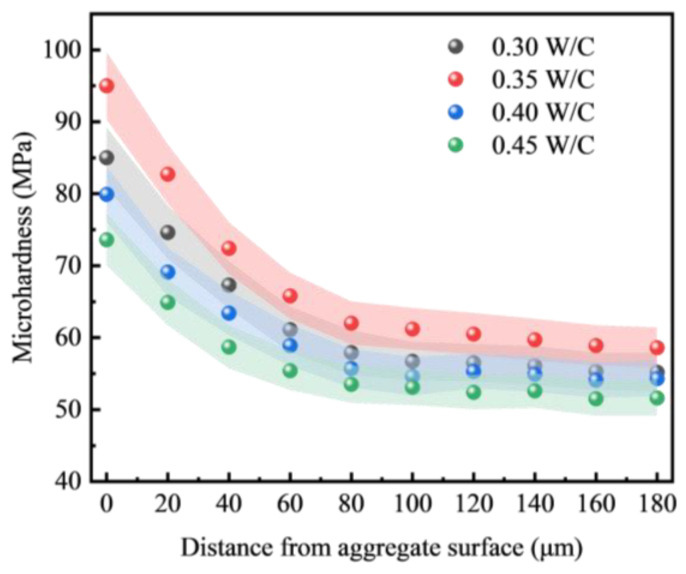
Effect of water–cement ratio on microhardness of interfacial transition zone.

Figure 12 illustrates the impact of the water–cement ratio on the microhardness of the interfacial transition zone (ITZ) in alkali-cured FULA concrete. The figure reveals that the microhardness of the ITZ in alkali-cured FULA concrete consistently surpasses that of the cement paste matrix, albeit with variations attributable to the water–cement ratio. Notably, as the water–cement ratio exceeds 0.35, the microhardness of the ITZ diminishes progressively with an increase in the water–cement ratio. This decrement is attributed to the rise in free water, which in turn diminishes the densification of the ITZ.

When the water–cement ratio falls below 0.35, a gradual decline in the microhardness of the interfacial transition zone (ITZ) is also observed as the ratio decreases further. This phenomenon is attributed to an excessively low water–cement ratio, which impairs the workability of the concrete paste. Consequently, it hinders the adhesion and hydration of cementitious materials on the aggregate surface during mixing. Notably, at a water–cement ratio of 0.35, the microhardness attains its peak value at a consistent distance from the aggregate surface, suggesting an optimal bonding state between the alkali-cured FULA and the cement stone matrix. This finding aligns with the compressive strength development pattern of concrete depicted in Figure 10.

#### 3.5.2. Micromorphology

**Figure 13:**
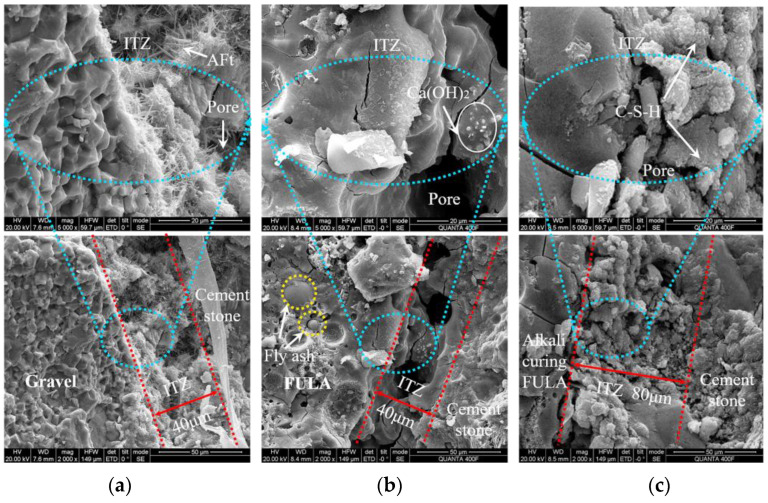
Microscopic morphology of the transition zone at the concrete interface. (**a**) Gravel concrete; (**b**) Ordinary FULA concrete; (**c**) Alkali curing FULA concrete.

Figure 13 illustrates the micro-morphology of the transition zone at the “coarse aggregate-cement stone” interface within a concrete specimen section. Examination of Figure 13a reveals that the interface transition zone in gravel concrete exhibits a loose and porous structure, characterized by the proliferation of numerous needle-like ettringite crystals. This underlying factor accounts for the reduced micro-hardness observed in the gravel concrete’s interfacial transition zone.

In contrast, Figure 13b depicts a microscopic morphology in which ordinary FULA and the cement stone matrix share greater similarity. Notably, the interfacial transition zone in ordinary FULA concrete exhibits enhanced density compared to crushed stone concrete. However, a significant quantity of unhydrated fly ash particles persists within the FULA, and the transition zone also contains numerous large pores. This observation suggests that the bond between the unsurfaced FULA and the cement stone matrix lacks tightness. The cause of this phenomenon lies in the low degree of hydration of fly ash particles on the FULA surface, impeding the formation of a strong bond with the cement paste.

Figure 13c showcases a striking contrast in microscopic morphology between alkali-cured FULA and the cement stone matrix. The cross-section of alkali-cured FULA is smooth and devoid of fly ash particles, indicating complete hydration of the fly ash. The pores within the interfacial transition zone are filled with abundant C-S-H gel, fostering the formation of an interwoven nested structure between FULA and the cement stone. Additionally, under the internal curing effect of FULA, the structure becomes even denser. Consequently, the microhardness of the interfacial transition zone surpasses that of the cement stone matrix, explaining the observed increase in hardness.

## 4. Mechanistic Analysis

### 4.1. Mechanism of Reinforcement of FULA by Alkali Curing

#### 4.1.1. Cementation Mechanism of Cement on FULA

**Figure 14:**
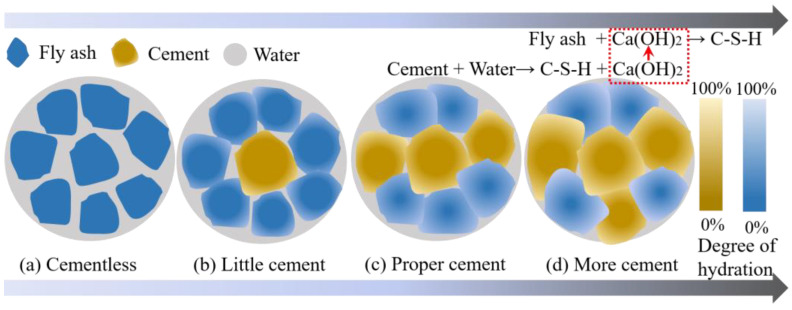
Cementation mechanism of cement to FULA.

Figure 14 delineates the cementation mechanism of cement on FULA. In the absence of cement (Figure 14a), fly ash exhibits minimal self-hardening capability, rendering it challenging to consolidate into a monolithic material possessing defined mechanical properties. Upon the introduction of a modest amount of cement, the hydration product, C-S-H gel, emerges as the primary cementing agent within the system. Additionally, fly ash contributes a secondary cementing effect, facilitated by the presence of Ca(OH)_2_ (Figure 14b). However, it is noteworthy that the quantities of both C-S-H gel and Ca(OH)_2_ produced are relatively limited, resulting in a lower degree of fly ash hydration and, consequently, reduced mechanical properties of FULA.

With the increase of cement content, the C-S-H gel and Ca(OH)_2_ produced by cement hydration increased. The densification of FULA as well as the hydration degree of fly ash are enhanced, resulting in the strength of FULA will also increase significantly (Figure 14c). However, Ca(OH)_2_ has a low solubility and has limited activation effect on fly ash. Continuing to increase the content of cement is not conducive to low carbon development nor to a significant improvement in the mechanical properties of FULA (Figure 14d). Therefore, a more effective alkaline environment needs to be provided for fly ash in order to further stimulate the potential activity of fly ash and improve the mechanical properties of FULA.

#### 4.1.2. Mechanism of Reinforcement of Mechanical Properties of FULA by Alkali Curing

**Figure 15:**
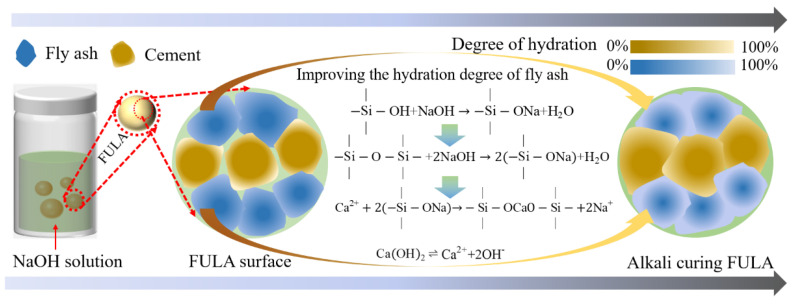
Mechanism of reinforcement of FULA by alkali curing.

Figure 15 elucidates the enhancement mechanism of alkali curing on the mechanical properties of FULA. Inspection of the figure reveals that, in an alkaline solution, the fly ash particles situated on the surface of FULA are the first to undergo reaction with sodium hydroxide. Under the erosive action of sodium hydroxide, the silica hydroxyl groups (-Si-OH) on the surface of these particles are disrupted and engage in an initial ion exchange with the solution. As the soaking duration extends, sodium hydroxide penetrates deeper into the interior of FULA, causing the rupture of silica-oxygen bonds (-Si-O-Si-) within the embedded fly ash. Throughout the process of sodium hydroxide erosion, the concentration of calcium ions, derived from the dissolution of calcium hydroxide (a hydration product of cement) in the curing solution, progressively increases. These calcium ions subsequently react with the previously disrupted silica hydroxyl groups, leading to the formation of hydrated calcium silicate. This sequence of reactions enhances the hydration degree of the fly ash and promotes the densification of FULA’s microstructure, ultimately resulting in a further augmentation of FULA’s mechanical properties.

The elemental calcium present in FULA primarily originates from cement, which undergoes hydration to form calcium hydroxide. The solubility product constant (Ksp) of calcium hydroxide is given by $K_{\mathrm{SP}}=\left[{\mathrm{Ca}}^{2+}\right]\cdot {\left[{\mathrm{OH}}^{-}\right]}^{2}=5.5\;\times \;10^{-6}$. In sodium hydroxide solutions, an increase in the concentration of OH^−^ results in a decrease in the concentration of Ca^2+^, and conversely. However, when sodium hydroxide is employed for the maintenance of FULA, the concentration of OH^−^ in the external solution surrounding the aggregate is substantially greater than that within its interior. Due to this concentration gradient, OH^−^ from the external solution continuously diffuses into the aggregate’s interior (as illustrated in Figure 15). This influx raises the OH^−^ concentration within the aggregate, causing a corresponding decrease in the Ca^2+^ concentration. Consequently, the reduced Ca^2+^ concentration leads to a decrease in the formation of hydrated calcium silicate, thereby slowing the early-stage strength development of alkali-cured FULA.

As the curing time progresses, despite the high OH^−^ concentration and low Ca^2+^ concentration in the system, Ca^2+^ continues to displace Na^+^ in -Si-ONa- structures, leading to the precipitation of hydrated calcium silicate. This ongoing displacement and precipitation process results in the continuous dissolution of new Ca^2+^ and the sustenance of the reaction, ultimately contributing to an increase in the late-stage strength of the fly ash lightweight aggregate. However, when the concentration of the sodium hydroxide conservation solution is excessively high, the reaction between sodium hydroxide and fly ash proceeds rapidly, forming a significant amount of reactive material around the particles. This hinders further reaction progression. Additionally, under highly alkaline conditions, the hydration product C-S-H gel becomes unstable and susceptible to degradation, leading to a decline in strength during the later stages.

### 4.2. Mechanism of Reinforcement of Alkali Curing on FULA Concrete Interfacial Transition Zone

#### 4.2.1. Mechanism of Different Aggregates on the Interfacial Transition Zone

The interfacial transition zone (ITZ) in conventional concrete exhibits high porosity and is characterized by a loose structure, enriched with an abundance of oriented needle-and-rod AFt and flaky Ca^2+^, making it the weakest component within the concrete matrix. This weakness arises from the smoother, denser stone surfaces that exhibit lower water absorption rates, leading to poor compatibility with cement paste and the formation of a prominent demarcation area at the contact surface during cement hydration. In contrast, during the mixing phase of lightweight aggregate concrete, cement paste can penetrate into the lightweight aggregates through their open pores and surface microcracks. The hydration products of the cement are embedded within the aggregates, fostering a tight, interlocking structure that effectively bridges the lightweight aggregates and cement paste.

**Figure 16:**
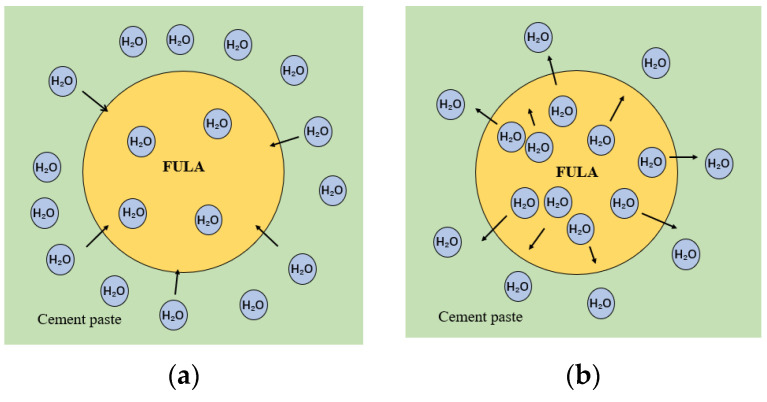
The role of internal maintenance of FULA. (**a**) Early stages of hydration; (**b**) Late stage of hydration.

During the initial stages of cement hydration, the cement paste contains an abundance of water (Figure 16a). The micropores on the surface of FULA absorb this excess moisture, mitigating the adverse effects of water seepage on the aggregate surface. Consequently, the contact area between the FULA and cement paste becomes denser. As cement hydration progresses to its later stages, water in the cement paste diminishes due to evaporation (Figure 16b). At this point, the FULA begins to release its internally stored water, enabling more complete hydration of the surrounding cement paste. However, the FULA’s water-releasing capacity diminishes with distance, causing the degree of hydration in the ITZ to decrease as one moves away from the lightweight aggregate surface. Consequently, the microhardness of the ITZ exceeds that of cementite, with microhardness increasing in proximity to the FULA surface.

#### 4.2.2. Mechanism of the Effect of Water–Cement Ratio on the Interfacial Transition Zone

With an augmentation in the water–cement ratio, the interfacial transition zone (ITZ) of lightweight aggregate concrete experiences more thorough hydration within the cement stone matrix. However, this enhancement in hydration also leads to an increase in cement stone porosity within the ITZ, thereby reducing the overall compactness of the concrete. As the hydration reaction progresses, the amount of free water available for cement hydration diminishes, resulting in a decrease in the relative humidity within the concrete. Notably, the reduction in relative humidity within lightweight aggregate concrete accelerates as the water–cement ratio decreases, thereby intensifying the humidity gradient between the lightweight aggregate and the cement stone in the ITZ. Consequently, moisture within the lightweight aggregate becomes more prone to diffusion into the cement stone, amplifying the internal curing effect of the aggregate. Therefore, a lower water–cement ratio correlates with a more pronounced internal curing effect from the lightweight aggregate, ultimately leading to enhanced microhardness in the ITZ of lightweight aggregate concrete.

## 5. Conclusions

(1) There is a positive correlation between the cement content and the numerical tube pressure and packing density of FULA. With the increase of cement content, the numerical tube pressure and packing density of FULA can be increased continuously. However, when the cement content is low, FULA is almost difficult to be molded. When the cement dosage is very high, FULA transforms to hardened cement stone, and the increase of numerical tube pressure and packing density slows down.
(2) There are large differences in the effects of different types of alkaline solutions on the basic properties of FULA. Sodium hydroxide solution has the effect of increasing the basic properties of FULA (numerical tube pressure, bulk density, 1 h water absorption). In addition, sodium sulfate, sodium chloride, and their complex solutions are detrimental to the development of FULA basic properties at any concentration.
(3) FULA after sodium hydroxide solution maintenance does not generate new substances, but the quartz minerals in the fly ash are significantly consumed, and the generation of hydration products C-S-H gel is significantly improved. However, the larger concentration of sodium hydroxide solution is not favorable to the generation of hydration products. The reason is that moderate amount of sodium hydroxide can promote the dissolution of fly ash, but excessive sodium hydroxide also has a dissolving effect on the hydration products.
(4) Compared with gravel concrete and ordinary FULA concrete, the microhardness of the interfacial transition zone of alkali curing FULA concrete is significantly improved. The microhardness of the interfacial transition zone of gravel concrete and ordinary FULA concrete is lower than that of the cement stone matrix, and the closer to the aggregate, the more obvious the microhardness decrease. However, the microhardness of the interface transition zone of alkali curing FULA concrete is significantly higher than that of the cement stone matrix, and the closer to the aggregate, the more obvious the microhardness increase.
(5) In this paper, FULA and its concrete with excellent performance were prepared by alkali curing method, but there are still more problems that need to be explored in depth. This paper focuses on the performance of short-term immersion FULA, and the change rule of mechanical properties of long-term immersion FULA is not yet understood. This paper focuses on the mechanical properties of FULA concrete, but the effect of FULA on the durability of concrete such as resistance to sulphate attack and carbonation is not involved.

## Figures and Tables

**Table 1:** The mineral composition of the cement (%).

Component	C_3_S	C_2_S	C_3_A	C_4_AF	Others
Content	54.04	22.84	8.39	10.42	4.31

**Table 2:** The Chemical composition of the cement (%).

Category	SiO_2_	A1_2_O_3_	Fe_2_O_3_	CaO	MgO	f-CaO	Na_2_O	Loss
Cement	23.48	5.36	3.43	65.10	2.11	0.39	0.47	0.13
Fly ash	58.13	32.75	1.14	1.09	0.93		0.94	5.02

**Table 3:** Mixing ratios of concrete with different aggregates (kg/m^3^).

Categories	D1	D2	D3
Cement	400	400	400
Coarse aggregate	1000	770	770
Fine Aggregate	550	550	550
Water	120	120	120
water reducer	0.5%	0.5%	0.5%
Coarse aggregate type	Gravel	Ordinary FULA	Alkali curing FULA

## Data Availability

The original contributions presented in the study are included in the article, further inquiries can be directed to the corresponding authors.

## Nomenclature

The definition of terms.

FULA	Fly Ash Unburned Lightweight Aggregate
ITZ	Interfacial Transition Zone
